# The Diabetes Mellitus–Atherosclerosis Connection: The Role of Lipid and Glucose Metabolism and Chronic Inflammation

**DOI:** 10.3390/ijms21051835

**Published:** 2020-03-06

**Authors:** Anastasia Poznyak, Andrey V. Grechko, Paolo Poggio, Veronika A. Myasoedova, Valentina Alfieri, Alexander N. Orekhov

**Affiliations:** 1 Institute for Atherosclerosis Research, Skolkovo Innovative Center, 121609 Moscow, Russia; tehhy_85@mail.ru; 2 Federal Scientific Clinical Center for Resuscitation and Rehabilitation, 109240 Moscow, Russia; noo@fnkcrr.ru; 3 Unit for the Study of Aortic, Valvular and Coronary Pathologies, Centro Cardiologico Monzino IRCCS, 20138 Milano, Italy; Paolo.Poggio@ccfm.it (P.P.); veronika.myasoedova@gmail.com (V.A.M.); valentina.alfieri@ccfm.it (V.A.); 4 Laboratory of Angiopathology, Institute of General Pathology and Pathophysiology, 125315 Moscow, Russia; 5 Institute of Human Morphology, 117418 Moscow, Russia

**Keywords:** atherosclerosis, diabetes mellitus, cardiovascular disease, chronic inflammation, lipid metabolism, hyperglycemia

## Abstract

Diabetes mellitus comprises a group of carbohydrate metabolism disorders that share a common main feature of chronic hyperglycemia that results from defects of insulin secretion, insulin action, or both. Insulin is an important anabolic hormone, and its deficiency leads to various metabolic abnormalities in proteins, lipids, and carbohydrates. Atherosclerosis develops as a result of a multistep process ultimately leading to cardiovascular disease associated with high morbidity and mortality. Alteration of lipid metabolism is a risk factor and characteristic feature of atherosclerosis. Possible links between the two chronic disorders depending on altered metabolic pathways have been investigated in numerous studies. It was shown that both types of diabetes mellitus can actually induce atherosclerosis development or further accelerate its progression. Elevated glucose level, dyslipidemia, and other metabolic alterations that accompany the disease development are tightly involved in the pathogenesis of atherosclerosis at almost every step of the atherogenic process. Chronic inflammation is currently considered as one of the key factors in atherosclerosis development and is present starting from the earliest stages of the pathology initiation. It may also be regarded as one of the possible links between atherosclerosis and diabetes mellitus. However, the data available so far do not allow for developing effective anti-inflammatory therapeutic strategies that would stop atherosclerotic lesion progression or induce lesion reduction. In this review, we summarize the main aspects of diabetes mellitus that possibly affect the atherogenic process and its relationship with chronic inflammation. We also discuss the established pathophysiological features that link atherosclerosis and diabetes mellitus, such as oxidative stress, altered protein kinase signaling, and the role of certain miRNA and epigenetic modifications.

## 1. Introduction

Atherosclerosis is a widespread chronic inflammatory disorder of the arterial wall that often leads to disability and even death. At its final stages, atherosclerosis manifests itself as a lesion of the intimal layer of the arterial wall and accumulation of plaques. Subsequent erosion or rupture of atherosclerotic plaques triggers thrombotic events that can potentially be fatal. Decades of intensive research made it clear that atherosclerosis has complex pathogenesis, the main components of which are lipid accumulation and chronic inflammation in the arterial wall [1]. Atherosclerosis is classically associated with altered lipid metabolism and hypercholesterolemia [2]. An elevated level of circulating modified low-density lipoprotein (LDL) is a known risk factor of cardiovascular diseases [3]. However, the disease pathogenesis appears to be more complex than lipid metabolism changes and involves multiple factors, the most prominent of which is inflammation [4].

The chain of pathological events that leads to atherosclerosis development is believed to be initiated by local endothelial dysfunction, which may be caused by blood flow turbulence near the sites of artery bends or bifurcations. The blood vessel endothelium responds to the mechanical stress with activation that subsequently leads to the recruitment of circulating immune cells. Circulating monocytes adhere to the injured area of the arterial wall and penetrate inside, differentiating into macrophages that actively participate in lipid uptake through phagocytosis and give rise to foam cells that are abundantly present in atherosclerotic plaques [5].

Detailed study of atherosclerotic lesion development is complicated by the fact that the process may differ considerably in humans and available model animals [6,7]. However, the main outlines of the process could be established. The early stage of atherosclerotic lesion development is known as “fatty streak”, an area in the vascular wall that is characterized by intracellular lipid accumulation by foam cells, which also contains vascular smooth muscle cells (VSMCs) and T lymphocytes. Fatty streaks can further progress to atherosclerotic lesions if chronic injury of the endothelium persists. In the growing lesions, intracellular lipid accumulation involves several cell types. The recruited macrophages internalize LDL particles via phagocytosis and contribute to the local production of inflammation mediators. Resident intimal cells also actively participate in this process. The stellar-shaped macrovascular pericytes form a three-dimensional cellular network in the subendothelial layer of the intima, forming contacts with each other and endothelial cells and ensuring tissue homeostasis. This network is disrupted in atherosclerotic plaques due to pericyte phenotypic changes, leading to a loss of intercellular contacts and increased production of extracellular matrix components [8]. VSMCs involved in the pathological process can also undergo a phenotypic switch, possibly acquiring proliferative and secretory properties [9]. At the later stages of the disease development, plaques can acquire a stable fibrous cap that separates them from the vessel milieu. Destabilization of the plaque occurs through depletion and rupture of the fibrous cap facilitated by matrix metalloproteinases (MMPs) that provoke extracellular matrix degradation. Macrophages and other inflammatory cells serve as important sources of these enzymes in the plaque [10]. The mechanisms responsible for plaque erosion need to be further investigated. These processes are especially difficult to model in atherosclerotic animals [6]. Inflammatory events, such as local platelet-mediated neutrophil activation, release of myeloperoxidase, toll-like receptor (TLR-2) signaling, and neutrophil-mediated injury, appear to play their roles in this process [11].

Atherosclerotic plaques can reduce the lumen of the blood vessel, leading to ischemia and metabolic changes in the alimented tissues [12]. Even more dangerous is thrombogenesis induced by unstable plaques and, in some cases, on the surface of undamaged plaques, which can often lead to fatal consequences [13].

## 2. Diabetes Mellitus

Diabetes mellitus is a group of disorders of carbohydrate metabolism, whose main feature is chronic hyperglycemia that results from defects of insulin secretion, insulin action, or a combination of those. Metabolic abnormalities observed in diabetes can be caused by the low level of insulin production and/or insulin resistance of the target tissues. The disease affects primarily skeletal muscles and adipose tissue, but also liver, at the level of insulin receptors, the signal transduction system, and/or effector enzymes or genes [14]. Symptoms of hyperglycemia include polyuria, polydipsia, weight loss, sometimes accompanied by polyphagia, and blurred vision. It can also be accompanied by growth impairment and susceptibility to certain infections. The direct life-threatening consequences of uncontrolled diabetes are hyperglycemia with ketoacidosis or the nonketotic hyperosmolar syndrome [15]. However, some patients, mostly with type 2 diabetes, can remain asymptomatic during the early years of the disease.

### 2.1. Type 1 Diabetes

Type 1 diabetes (T1D) is caused by the autoimmune destruction of insulin-producing pancreatic β-cells [16]. The classic trio of T1D symptoms is polydipsia, polyphagia, and polyuria. Most often the disease is diagnosed in children and adolescents, who usually demonstrate the abovementioned combination of symptoms and a marked hyperglycemia that necessitates lifelong exogenous insulin replacement. The study of T1D pathogenesis was mostly based on two animal models of the disease: the nonobese diabetic mouse and the BioBreeding-diabetes-prone rat, both of which are characterized by progressive T-cell-mediated destruction of β-cells [17]. However, the differences between rodent models and the human situation limited the transferability of the obtained results. In humans, autoantibodies were present in 70–80% of patients at the time of diagnosis [18]. Immunosuppressive and immunointerventive approaches for preventing T1D did not result in preservation of β-cell function or acted only temporally [19,20].

Patients with T1D develop pancreatic lesions that can lead to acute pancreatitis with leukocyte accumulation [17]. The disease affects both exocrine and endocrine components of the pancreas.

### 2.2. Type 2 Diabetes

Increasing prevalence of type 2 diabetes mellitus (T2D), which now affects more than 370 million people, is a result of the worldwide increased incidence of obesity. Diabetes is diagnosed from fasting and two-hour glucose levels following a standardized oral glucose load. Prediabetes is often determined as the distinction between impaired fasting glucose and/or impaired glucose tolerance [21]. However, T2D should probably be regarded as a continuum of disease stages with increasing severity, in which the degree of plasma glucose increase relies on the magnitude of β-cell deficiency. Insulin resistance is already well established when impaired glucose tolerance is present and the increase in glucose, even across the normal range, is due to a continuous decline in β-cell function [22]. The disease was shown to be heritable in some cases, and individuals with first-degree relatives affected by diabetes are at increased risk of its development [23]. It was demonstrated that β-cell function is heritable [24] and that β-cell function crucially determines glucose intolerance and T2D in different racial and ethnic groups [25]. It is currently known that the pathogenesis of T2D is heterogeneous, and processes other than insulin resistance and β-cell dysfunction are involved in its development.

## 3. Diabetes Mellitus and Atherosclerosis: Pathophysiological Conjunction

**Figure 1:**
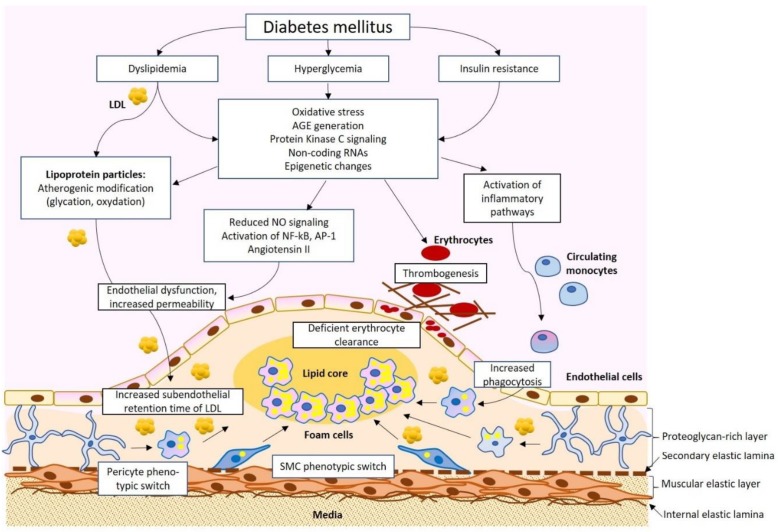
A simplified scheme of the pathophysiological connection of diabetes mellitus and atherosclerosis. Dyslipidemia, hyperglycemia, and insulin resistance result in a spectrum of physiological changes, including the formation of atherogenic low-density lipoprotein (LDL), advanced glycation end products (AGE), and activation of pro-inflammatory signaling that impact different cell types of the arterial wall, resulting in atherosclerotic lesion development. SMC, smooth muscular cells.

Diabetes mellitus and atherosclerosis appear to be connected through several pathological pathways. Increased risk and accelerated development of atherosclerosis have been shown in studies on diabetic patients. For instance, several studies have reported the early development of atherosclerosis in adolescents and children with T1D [26,27]. Among the factors explaining such acceleration, dyslipidemia with increased levels of atherogenic LDL, hyperglycemia, oxidative stress, and increased inflammation have been proposed (Figure 1).

### 3.1. Dyslipidemia in Diabetic Patients

One of the most studied links between diabetes and atherosclerosis is the level of small dense LDL (sdLDL), which is a known risk factor of atherosclerosis. Although LDL is currently recognized as the main source of intracellular lipid accumulation in the plaque, native LDL particles do not cause prominent lipid accumulation in cultured cells; hence, they do not possess atherogenicity. It is atherogenic modification of LDL, which alters the physical–chemical characteristics of LDL particles, that triggers massive lipid accumulation [3]. A cascade of multiple modifications of LDL has been proposed as a plausible model for LDL atherogenic modification in the blood. According to this model, an LDL particle first becomes desialylated, which is followed by the increase of particle density, decrease of its size, and acquisition of negative charge [28]. Such particles can be isolated based on their physical properties. Very-low-density LDL (VLDL) is another subfraction of altered LDL described in humans. Small dense lipoprotein particles are more susceptible to oxidation because of the reduced antioxidant content and altered lipid composition. It is likely that oxidation takes place at the last stages of atherogenic LDL modification [29,30].

After penetration into the subendothelial space at the atherosclerotic lesion site, modified LDL particles reside there for a longer time due to interaction with proteoglycans and therefore have increased chances of being internalized by the lesion cells. Moreover, modified LDL has a lower affinity to LDL receptor (LDLR) and is therefore internalized mainly through unspecific phagocytosis, which leads to intracellular cholesterol accumulation rather than normal degradation of lipoprotein particles. These processes result in the formation of foam cells with the cytoplasm filled with accumulated lipid droplets [31].

Studies conducted in a murine model of diabetes clearly demonstrated the significance of LDL modification for the increase of the subendothelial retention time of lipoprotein particles. The authors extracted the LDL fraction from the blood of T1D patients and healthy controls, injected it into diabetic mice, and measured its retention in the atherosclerosis-prone areas of the arterial wall. It turned out that retention of LDL obtained from T1D patients was more than fourfold higher than that of LDL obtained from control subjects [32].

A cross-sectional study revealed altered lipoprotein levels in young adolescents with T1D and T2D. Increased concentrations of apolipoprotein B, sdLDL, and LDL-cholesterol were not highly prevalent in young subjects with T1D but had increased prevalence in subjects with T2D [33]. Importantly, studies that did not assess atherogenic LDL subfractions and only looked at the total level of circulating LDL may have missed this risk factor in diabetic patients, since the latter may remain within the normal range even when sdLDL is abnormally elevated.

Epidemiological studies indirectly demonstrated the effect of T1D- and T2D-associated metabolic changes on atherosclerosis development. For instance, the Diabetes Control and Complications Trial in patients with T1D and the United Kingdom Prospective Diabetes Study in patients with T2D demonstrated that patients on the conventional treatment who did not achieve adequate blood glucose control were at higher risk of vascular complications than those receiving intensive treatment to ensure strict glucose control [34,35].

Diabetes-associated dyslipidemia received much attention during recent years and became the subject of numerous review papers [36,37]. Alteration of the blood lipid profile in diabetes is linked to elevated hepatic production of triglyceride-rich lipoproteins, leading to the increased formation of atherogenic VLDL. This alteration can partially be corrected by insulin treatment.

### 3.2. The Role of Hyperglycemia and Advanced Glycation End-Products in Atherosclerosis

The risk of diabetic cardiovascular complications after exposure to high glucose levels for a certain period of time is called “metabolic memory” or “legacy effect”. One of the possible mechanisms of this effect is the formation of advanced glycation end-products (AGE), which occurs when the blood glucose level is high. These compounds are not easily metabolized, accumulating in patients with a long history of inadequate blood glucose control. Such accumulation may accelerate the progression of vascular disease in diabetic patients. Numerous studies have established the link between inadequately controlled blood glucose level and microvascular complications of diabetes, such as renal and retinal symptoms. However, the relationship between elevated blood glucose and atherosclerosis of large arteries appears to be less straightforward. Direct pro-atherogenic effects of glucose levels on the cell types typically present in atherosclerotic lesions could not be demonstrated [38]. It remains possible that elevated glucose acts primarily on tissues, including liver or adipose tissue, and the effect on atherosclerotic lesion cells is mediated by altered signaling from these tissues. An elevated intracellular glucose level increases the flux through cellular metabolic pathways, such as the mitochondrial electron transport system, which may result in reactive oxygen species (ROS) overproduction. Moreover, glucose metabolites can induce pro-inflammatory responses through activation of protein kinase C-beta and aldose reductase [39].

Another possibility is that elevated glucose acts primarily through extracellular mechanisms, for example, by inducing glycation and glycoxidation of proteins, resulting in AGE formation. AGE accumulate in diabetic patients when the blood glucose level is elevated and appear to play an important role in atherosclerosis development. These molecules influence endothelium activation and surface expression of adhesion molecules, thereby promoting the adhesion and entrance of monocytes/macrophages into the subendothelial space during the initial stages of plaque formation. Moreover, these molecules enhance cytokine release by macrophages, thereby maintaining a pro-inflammatory context within the developing plaque. Another mechanism is glycation of LDL particles, which can be regarded as one of the atherogenic modifications of LDL. It was also shown that AGE may inhibit reverse cholesterol transport by reducing the expression of ATP-binding membrane cassette transporters A1 and G1 (ABCA1 and ABCG1) on monocytes, to enhance vasoconstriction by increasing endothelin-1 levels, and to reduce vasodilation by decreasing nitric oxide levels. Finally, AGE participate in the modification of extracellular matrix molecules, which also promotes atherosclerotic lesion development [40,41]. Modification of the extracellular matrix proteins by excessive glycation promotes interaction with AGE receptor RAGE on macrophages, endothelial cells, VSMCs, and other cell types. Such interaction results in pro-inflammatory effects and increased intracellular ROS generation [42].

In mouse models of atherosclerosis, such as apolipoprotein E-deficient (*apoE^-/-^*) mice with chemically induced diabetes, RAGE deficiency was shown to alleviate atherosclerotic lesion development [43]. These findings open the possibility of using RAGE inhibition for reducing atherosclerosis development in diabetic patients.

The direct role of AGE in stimulating the expression of scavenger receptors and promoting phagocytosis has been revealed in a recent study [44]. In this study, AGE-modified bovine serum albumin induced morphological changes in cultured murine macrophages, increasing their phagocytic activity. This effect was attenuated by a fucose-containing sulphated polysaccharide, fucoidan, which has known anti-inflammatory properties. Diabetes is known to be associated with a pro-inflammatory state, which is discussed below in more detail. It is possible that enhanced glucose uptake by lesional cells is promoted by the pro-inflammatory signaling and increased phagocytic activity by lesion macrophages rather than the direct effect of hyperglycemia.

Further insights on possible links between hyperglycemia and atherosclerosis came from animal studies. Studies of hyperglycemia’s effect on vascular lesions in the *apoE^-/-^* mouse model revealed that advanced lesions appear in hyperglycemic mice earlier than they do in normoglycemic controls. Moreover, accelerated atherogenesis was observed earlier than any detectable divergence in the plasma lipid parameters in normoglycemic mice [45]. A new model of hyperglycemia-accelerated atherosclerosis was created by crossing *apoE^-/-^* or LDLR-deficient mouse strains with mice carrying a point mutation in the gene encoding insulin (Ins2^+/Akita^:*apoE^-/-^* mice) [45]. These animals were characterized by spontaneous development of diabetes and atherosclerosis, presenting with insulin deficiency, hypercholesterolemia (predominantly through LDL-cholesterol increase), and accelerated formation of atherosclerotic plaques while kept on a regular chow diet. The authors reported deficient lipoprotein clearance through lipolysis-stimulated lipoprotein receptors and altered lipoprotein composition. This animal model was expected to be useful for studying atherosclerosis in the context of T1D and testing possible therapeutic approaches. For instance, Ins2^+/Akita^:*apoE^-/-^* mice were used to demonstrate the beneficial effect of leptin on atherosclerotic plaque progression [46].

Excessive glycation may also play a role at later stages of atherosclerosis development. As demonstrated in a recent study, glycation of erythrocytes in T2D patients may promote their internalization by the endothelial cells via phagocytosis, which impairs endothelial function. This process is likely to contribute to unstable plaque development with subsequent thrombosis in patients with T2D and atherosclerosis [47].

The level of AGE may also be used for diagnostic purposes to assess the risk of atherosclerosis development and vascular complications in diabetic patients. In a recent study, measurement of skin AGE levels through autofluorescence (AF) in Japanese T1D patients and their gender- and age-matched healthy controls demonstrated the increased AF in diabetes that appeared to be an independent risk factor for carotid atherosclerosis [48].

### 3.3. The Role of Oxidative Stress

Diabetes is known to be associated with both increased ROS production and reduced activity of antioxidant systems [49]. Studies in vitro have demonstrated that increased ROS production is linked to hyperglycemia [50]. Further studies in animals have revealed the involvement of NADPH oxidase family protein Nox1, which was up-regulated in diabetic mice. Knockdown of this protein alleviated atherosclerosis progression in such animals [51]. The role of oxidative stress in diabetes-associated atherosclerosis was confirmed in experiments on *apoE^-/-^* mice deficient for one of the main regulators of antioxidant enzymes, glutathione peroxidase 1 (Gpx1). Upon diabetes induction with streptozotocin, animals that were also deficient for Gpx1 had accelerated atherogenesis, with increased plaque size, macrophage infiltration, and increased expression of inflammatory markers, while restoration of Gpx1 reduced atherogenesis [52]. Overall, vascular ROS increase appears to be closely related to atherosclerosis in the diabetic context, and antioxidant therapies may still be considered for the management of the disease, although more selective approaches are needed to achieve relevant results with antioxidant drugs [40].

### 3.4. The Role of Protein Kinase C (PKC) Activation

Protein kinase C (PKC) is one of the key protein kinases mediating the cellular signaling pathway, which responds to cytokines, growth factors, and other messenger molecules [53]. Increased glucose uptake by vascular cells results in increased synthesis of diacylglycerol, which is an activator of PKC. Enhanced PKC activation can also result in response to oxidative stress [54]. Increased vascular PKC activation was confirmed in animal models of diabetes. Enhanced PKC signaling has numerous pro-atherogenic effects, including reduced production of NO and impaired vasodilation, endothelial dysfunction and increased permeability, and increased production of cytokines and extracellular matrix [42]. The complexity of intracellular signaling cascades activated by PKC makes it difficult to pinpoint the exact mechanism of its pro-atherogenic effect. However, studies in *apoE^-/-^* mice have shown that chemical or genetic inhibition of PKCβ led to reduced formation of atherosclerotic lesions [55]. Another study conducted in *apoE^-/-^* and in *apoE^-/-^* and *PKCβ^-/-^* double knock-out mice with chemically induced diabetes showed that increased PKCβ activation was linked to accelerated atherosclerosis development through the induction of CD11c and pro-inflammatory activation of macrophages. Correspondingly, inhibition of PKCβ reduced the size of atherosclerotic lesions [56]. These findings indicate that PKC may be considered as a potential therapeutic target.

### 3.5. Diabetes-Associated Chronic Inflammation and Atherosclerosis

Chronic inflammation is a known feature that is common to both atherosclerosis and diabetes mellitus. Atherosclerosis is currently regarded as a chronic inflammatory condition. In patients with T2D, increased activity of inflammasomes and elevated levels of nucleotide-binding oligomerization domain-like receptor 3 (NLRP3) were demonstrated, together with increased levels of pro-inflammatory cytokines interleukin (IL)-1β and IL-18 [57]. One of the direct links between atherosclerosis and diabetes identified within the inflammatory pathways is neutrophil extracellular trap activation, or NETosis, a special type of cell death of macrophages, during which the cells release chromatin into the extracellular space to trap and kill bacteria. This process is known to be elevated in chronic sterile inflammation and autoimmune conditions where it contributes to pathology development [42]. Increased levels of NETosis markers were found in patients with T2D [58]. Moreover, it was shown that NETosis may be enhanced in hyperglycemic conditions [59]. The possible role of enhanced NETosis in atherosclerosis development was confirmed in animal models. The atherosclerotic *apoE^-/-^* mice that also lacked neutrophil elastase and proteinase-3 necessary for NETosis had reduced atherosclerotic lesion formation compared with single knock-out animals [60].

Naturally, an active search for anti-inflammatory drugs that could reduce the risk of atherosclerotic cardiovascular disease in diabetic patients was conducted during recent years [54]. Among the anti-inflammatory drugs used to treat diabetic patients are salicylates, which were shown to reduce the glucose level while being effective for cardiovascular disease prevention and reducing the risk of thrombosis [61]. The use of inflammatory cytokine inhibitors appeared to be a promising approach for reducing cardiovascular risk in diabetic patients. It was shown that canakinumab, a monoclonal antibody that binds and neutralizes IL-1β, significantly reduced markers of inflammation in patients with controlled diabetes mellitus and high cardiovascular risk, but had no major effect on LDL-cholesterol [62]. Another study showed that canakinumab had a similar beneficial effect for reducing cardiovascular risk in patients with and without diabetes, but had no effect on de novo diabetes incidence [63]. The search for effective anti-inflammatory therapies reducing atherosclerosis in diabetic patients continues [64].

### 3.6. The Role of Circulating Non-Coding RNAs

Non-coding RNAs were shown to be implicated in numerous human disorders and are currently regarded as possible biomarkers and disease modifiers. Advances in genetic methods allowed non-coding RNAs to be studied in more detail and revealed their associations with pathogenic processes. MicroRNA (miRNA) are short RNA fragments that can inhibit the expression of certain genes at the mRNA level. These RNA fragments can be produced by multiple cell types and tissues and can be found circulating in the blood either free or confined in membrane microvesicles. Accumulating evidence highlights miRNAs as important possible biomarkers. However, the complexity of the miRNA landscape associated with human diseases, including diabetes, is so high that it is probably more promising to study miRNA signatures (combinations of multiple miRNA) rather than single miRNA types [65].

In humans, more than 2500 miRNAs have been identified, and several of them were shown to play a role in diabetes mellitus pathogenesis. In particular, multiple miRNAs were found to be involved in the development of microvascular complications of diabetes [66]. Among the miRNA which are relevant for atherosclerosis as well as diabetes, miR-146 and miR-126 have received much attention. miR-146a and miR-146b play an important role in the endothelial cells, where their expression is induced by inflammatory cytokine signaling and serves as a negative feedback loop to control inflammatory endothelial activation [67]. Therefore, regulation of these miRNAs is likely to be implicated at the initial stages of atherosclerotic lesion development in diabetic patients. miR-126 expression was shown to be a risk factor of T2D development, and this miRNA played a protective role in a mouse model of atherosclerosis [68,69].

Another important miRNA, miR-378a, was shown to play an important role in the regulation of metabolism, including energy and glucose homeostasis [70]. A very recent study implicated this miRNA in atherosclerosis development. The authors showed that miRNA-378a targets signal regulatory protein alpha (SIRPa), thereby regulating phagocytosis and polarization of macrophages. Moreover, the level of this miRNA was reduced in the aorta of *apoE^-/-^* mice in comparison to controls, highlighting its important role in the regulation of atherosclerosis-associated processes [71].

Another non-coding RNA that likely plays a role in diabetes-associated atherosclerosis is long non-coding RNA Dnm3os (dynamin 3 opposite strand). This RNA was shown to be increased in macrophages from diabetic mice, including *apoE^-/-^* diabetic mice, as well as in monocytes from T2D patients. Overexpression of this RNA promoted inflammatory gene expression and phagocytosis by macrophages and led to chromatin epigenetic changes, further promoting the inflammatory response [72]. More studies are needed to identify and characterize relevant non-coding RNAs that may have detrimental or beneficial effects on cardiovascular risk in diabetic patients.

### 3.7. The Role of Epigenetic Mosdification

Both persistent and temporal hyperglycemic exposure were shown to influence several significant cellular signaling pathways, including PKC activation and oxidative stress, described above, and transforming growth factor (TGF)-β-SMAD-MAPK signaling [73,74]. Moreover, hyperglycemia was shown to enhance the flux into the polyol and hexosamine pathways and increase the formation of AGE that are also associated with alterations in signaling pathways [75,76]. All these various effects make hyperglycemia a prominent risk factor for diabetic complications and vascular events. Chromatin changes play an important regulative role in the establishing of the link between glycemia and vascular complications.

Hyperglycemia is associated with the range of chromatin modifications that affect the genetic signature of vascular endothelial cells. For example, a genome-wide sequencing study of aortic endothelial cells exposed to a high level of glucose revealed histone H3K9/K14 hyperacetylation patterns that were inversely associated with DNA methylation in CpG clusters. This finding correlates with the activation of transcription of pathways linked to atherogenic effects and vascular diseases. The study demonstrated that hyperglycemia can induce epigenetic changes in the vascular endothelium that are relevant for atherosclerosis development, thereby providing another link between diabetes and atherosclerosis pathogenesis [77].

Transient hyperglycemia also triggers mono-methylation of H3 histones at lysine 4 (H3K4m1) and other histone lysine modifications. H3K4m1 was shown to be written by the Set7 lysine methyltransferase. The observed changes at the promoter of the *RELA* gene encoding the NF-κB-p65 subunit persisted for 5–6 days after the cells were returned to a normoglycemic state [78,79]. Thus, cytokines, chemokines, and adhesion molecules are affected by hyperglycemia through the regulation of one of the crucial pro-inflammatory transcription factors related to vascular and metabolic complications, among which is atherosclerosis [80]. Among these molecules, vascular cell adhesion molecule 1 (VCAM-1), which promotes adhesion of monocytes to the arterial endothelial cells, and monocyte chemoattractant protein 1 (MCP-1), responsible for macrophage infiltration, should be highlighted [81]. The enhanced expression of both NF-κB-dependent MCP-1 and VCAM-1 and genes encoding NFκB-p65 itself was observed in aortas of apolipoprotein A knockdown mice that were previously exposed to hyperglycemia.

The concluding result of these hyperglycemia-induced changes is the transcriptional activation of genes that are related to endothelial dysfunction [82]. Acetylation, as well as hyperacetylation, is also possible and can lead to the enhanced expression of the following genes relevant for atherosclerotic lesion development at different stages through the inflammatory response and extracellular matrix degradation: *MCP-1*, *MMP10*, *ICAM*, *HMOX1*, and *SLC7A11* [76].

Further research identified the role of Set7/9, which possibly coactivates NF-κB transcriptional activity in monocytes in response to inflammation through the activation of the H3K4me promotor, and the analogous effect was observed in endothelial cells in response to hyperglycemia [80,81]. Moreover, the induction of Set7-mediated up-regulation of *HMOX1* was shown to be beyond the methylation of histones but linked to hyperglycemia [78]. It was also shown that early intrusive monitoring of the glycemic profile in diabetic patients can play an important role in preventing vascular complications. This indicates the important role of hyperglycemia in the long-term outlook, leading to a phenomenon called “metabolic memory” [82].

All the aforementioned findings suggest numerous links between hyperglycemia, epigenetic changes, and cardiovascular risk that cannot be ignored. However, there are still many blind spots in the understanding of the underlying molecular mechanisms and their connections.

## 4. Conclusions

Both types of diabetes mellitus have been shown to be independent risk factors for accelerated atherosclerosis development. It is now clear that the pathogenesis of diabetes mellitus and atherosclerosis are closely linked, but the mechanisms and molecular interactions of this linkage are still under discussion. Among the known pathological mechanisms connecting diabetes and atherosclerosis are dyslipidemia, hyperglycemia with AGE production, increased oxidative stress, and inflammation. Despite the continuing search for novel therapeutic approaches, few medications have shown strong beneficial effects with regard to reducing the risk of atherosclerosis development in the specific population of diabetic patients. Adequate glycemic control and reduction of known risk factors remain the most frequently used strategies for protecting such patients. More studies are needed to reveal the exact signaling mechanisms of diabetes-associated macrovascular damage and to identify specific therapeutic targets.
